# Sex and age interaction in fundamental circulatory volumetric variables at peak working capacity

**DOI:** 10.1186/s13293-021-00409-9

**Published:** 2022-01-03

**Authors:** Candela Diaz-Canestro, David Montero

**Affiliations:** 1 Libin Cardiovascular Institute of Alberta, University of Calgary, Calgary, Canada; 2 Cumming School of Medicine, Libin Cardiovascular Institute of Alberta, Faculty of Kinesiology, University of Calgary, 2500 University Drive NW, Calgary, AB Canada; 3 Faculty of Kinesiology, University of Calgary, Calgary, Canada

**Keywords:** Stress cardiac imaging, Intravascular volumes, Aerobic capacity, Female sex, Advanced age

## Abstract

**Background:**

Whether the fundamental hematological and cardiac variables determining cardiorespiratory fitness and their intrinsic relationships are modulated by major constitutional factors, such as sex and age remains unresolved.

**Methods:**

Transthoracic echocardiography, central hemodynamics and pulmonary oxygen (O_2_) uptake were assessed in controlled conditions during submaximal and peak exercise (cycle ergometry) in 85 healthy young (20–44 year) and older (50–77) women and men matched by age-status and moderate-to-vigorous physical activity (MVPA) levels. Main outcomes such as peak left ventricular end-diastolic volume (LVEDV_peak_), stroke volume (SV_peak_), cardiac output (*Q*_peak_) and O_2_ uptake (VO_2peak_), as well as blood volume (BV), BV–LVEDV_peak_ and LVEDV_peak_–SV_peak_ relationships were determined with established methods.

**Results:**

All individuals were non-smokers and non-obese, and MVPA levels were similar between sex and age groups (*P* ≥ 0.140). BV per kg of body weight did not differ between sexes (*P* ≥ 0.118), but was reduced with older age in men (*P* = 0.018). Key cardiac parameters normalized by body size (LVEDV_peak_, SV_peak_, *Q*_peak_) were decreased in women compared with men irrespective of age (*P* ≤ 0.046). Older age per se curtailed *Q*_peak_ (*P* ≤ 0.022) due to lower heart rate (*P* < 0.001). In parallel, VO_2peak_ was reduced with older age in both sexes (*P* < 0.001). The analysis of fundamental circulatory relationships revealed that older women require a higher BV for a given LVEDV_peak_ than older men (*P* = 0.024).

**Conclusions:**

Sex and age interact on the crucial circulatory relationship between total circulating BV and peak cardiac filling, with older women necessitating more BV to fill the exercising heart than age- and physical activity-matched men.

**Supplementary Information:**

The online version contains supplementary material available at 10.1186/s13293-021-00409-9.

## Background

Notwithstanding the undeniable complexity of the human body, the vital organ par excellence entails a remarkable mechanistic simplicity in its most fundamental function [1, 2]. The direct relationship between cardiac filling and stroke volume (SV), known as the ‘law of the heart’ or Frank–Starling mechanism, was originally formulated at the dawn of twentieth century [3]. In essence, the more the heart is filled, the greater the output (*Q* = SV × heart rate), until a plateau is reached [1, 2]. In humans, experimental evidence denotes that cardiac filling in the upright position is far beneath the plateau section, such that any change in left ventricular end-diastolic volume (LVEDV) alters SV proportionally [4]. In the absence of major cardiovascular abnormalities, cardiac filling primarily depends on the filling level of the circulatory system, i.e., blood volume (BV) [5]. BV modulates cardiac function at rest as well as at peak exercise, in which peak SV (SV_peak_) exclusively determines cardiac output (*Q*_peak_), since peak heart rate (HR_peak_) is essentially uniform for a given age [6]. Importantly, outstanding levels of cardiorespiratory fitness, as represented by *Q*_peak_ and its pulmonary correlate peak oxygen uptake (VO_2peak_), are strongly associated with large reductions in cardiovascular and overall mortality [7, 8]. Invariably, high levels of cardiorespiratory fitness are accompanied by BV expansion [9, 10]. Conversely, blood withdrawal induces proportional decrements in *Q*_peak_ and VO_2peak_, which do not recover until intravasscular volumes are restored [10–12], firmly supporting the crucial role of BV in the human circulatory system.

The essential physiology of the circulatory system appears to be fairly straightforward. Yet, the fact that our current understanding is based on animal experiments and human studies mainly comprising healthy young men cannot be overlooked [13–15]. Moreover, the remarkable cardiovascular phenotypic adaptability to abnormal hemodynamic conditions, lifestyle stimuli as well as constitutional factors should be considered [16–19]. Most notably, whether the constitutional factors per excellence, i.e., sex and age, alter the fundamental circulatory relationships remains unresolved [16, 19, 20]. As a case in point, the inexorable age-dependent stiffening and remodeling of the cardiovascular system may interact with sex-specific hemodynamic and hematological characteristics [16, 17, 21–23], plausibly altering the regulation of and relationship between primary volumetric variables in the systemic circulation [18, 24, 25]. While the prevailing emphasis on molecular cardiology is an ineluctable consequence of technological prowess, sound integrative bases of cardiovascular physiology have yet to be elucidated in a large fraction of the human population.

The purpose of the present study was to comprehensively assess the impact of sex and age on the primary physiological determinants of cardiorespiratory fitness and their intrinsic circulatory relationships. To this end, transthoracic echocardiography, central hemodynamics, pulmonary O_2_ uptake at rest and during submaximal and peak exercise, as well as intravascular volumes were assessed in healthy young and older women and men matched by age group and physical activity levels.

## Patients and methods

### Study participants

Eighty-five adult women and men matched by age group (young: ≤ 44 years; older: ≥ 50 years) and moderate-to-vigorous physical activity (MVPA) levels were recruited via advertisements on community notice boards in the city of Calgary. Inclusion criteria comprised healthy status, absence of current medical symptoms or medication limiting incremental exercise testing, and no history of cardiac, pulmonary or neuromuscular diseases. Individuals fulfilling the above criteria but having donated blood within 3 months prior to the study were excluded. The study was approved by the Conjoint Health Research Ethics Board (REB18-1654) of the University of Calgary and conducted in accordance with the declaration of Helsinki. Prior to the start of the experiments, informed oral and written consents were obtained from all participants.

### Experimental design

Participants were required to report once to our laboratory for testing in addition to a voluntary familiarization visit. All individuals were instructed to avoid strenuous exercise, alcohol and caffeine from 24 h prior to testing, as well as to maintain their usual baseline activity and daily dietary habits throughout the study. All measurements were performed after a fasting period (≥ 5 h) in a quiet room with controlled temperature between 22 and 23 ℃. Prior to testing the participants completed demographic and clinical questionnaires and rested in supine position for 20 min in order to stabilize cardiovascular, hemodynamic and hematological variables. MVPA levels were determined from established questionnaires as previously described [26].

## Experimental measures

### *Hemoglobin mass* (*Hb*_*mass*_) and blood volumes

Hemoglobin mass (Hb_mass_) was determined using the classic carbon monoxide (CO) rebreathing technique integrated in a semi-automated system with a very low typical error of measurement (TE ≤ 1.2%), as previously described [10, 27, 28]. In brief, following 20 min of supine rest, 2 ml of blood (baseline) was sampled from the median cubital vein via a 20-G venflon (BD, USA) and analyzed immediately in duplicate for percent carboxyhemoglobin (%HbCO), hemoglobin (Hb) concentration and hematocrit (Hct) (ABL80, Radiometer, Denmark). After the exercise test, individuals breathed 100% O_2_ for 4 min to flush nitrogen from the airways. After closing the O_2_ input, a bolus of 1.5 ml/kg of 99.5% chemically pure CO (Air Liquide, Canada) was administrated into the breathing circuit. Individuals rebreathed this gas mixture for 10 min. Then, an additional 2 ml blood sample was obtained and analyzed in duplicate as aforementioned. The change in %HbCO is used to calculate Hb_mass_, taking into account the small amount of CO that remains in the rebreathing circuit at the end of the procedure. Total red blood cell volume (RBCV), plasma volume (PV) and blood volume (BV) were determined from Hb_mass_, baseline Hb concentration and Hct, and normalized by body weight [10, 27, 28].

### Transthoracic echocardiography and central hemodynamics

Apical four-chamber and two-chamber cine-loops were recorded via high-resolution ultrasound (Mindray Medical M9, USA) and analyzed offline (TOMTEC Imaging Systems, Royal Philips, Netherlands) at rest, at a given submaximal workload (100 W), and at peak heart rate (HR_peak_) during incremental exercise. Following the American Society of Echocardiography and the European Association of Cardiovascular Imaging recommendations, cardiac chamber quantification was performed using the modified Simpson method (biplane method of disks) by tracing the endocardial border in both apical four-chamber and two-chamber views at end-diastole and end-systole (Additional file 1: Fig. S2) [29, 30]. Diastolic function was assessed via transmitral inflow velocities determined by pulsed-wave Doppler, with the sample volume placed between the mitral leaflet tips in the apical four-chamber view. The peak inflow velocity during early (*E*) and late (*A*) diastole were assessed, and the *E*/*A* velocity ratio was calculated. Moreover, tissue Doppler measurements were performed in the apical four-chamber view, with a 3-mm pulsed Doppler sample volume placed at the level of septal mitral annulus and lateral tricuspid annulus. Early (*e*’) and late (*a*’) peak diastolic annular velocities, peak systolic annular velocity (S’), myocardial isovolumic contraction time (IVCT) and isovolumic relaxation time (IVRT) were determined [29, 30]. In parallel, systolic blood pressure (SBP), diastolic blood pressure (DBP) and mean blood pressure (MBP) at the heart level were continuously assessed non-invasively via Finometer PRO (Finapres Medical Systems, Netherlands), with data exported into a pre-established acquisition software (Labchart 7, AD Instruments, UK). SV was determined as LVEDV minus left ventricular end-systolic volume (LVESV), while the product of SV and HR provided cardiac output (*Q*). Systemic vascular resistance (SVR) was calculated as the ratio of MBP and *Q*. Echocardiographic variables are commonly presented normalized by body surface area (BSA = 0.007184 weight^0.425^ height^0.725^) to attenuate the impact of body size on sex comparisons [31], except for intrinsic volumetric relationships within the circulatory system (assessed separately in each sex). The reproducibility of key echocardiographic and hemodynamic measurements (within-subject coefficient of variation (CV)) during incremental exercise in our laboratory is ≤ 6% for left ventricular (LV) volumes and ≤ 3% for blood pressures.

### Aerobic capacity

An established incremental exercise protocol [10, 27] was implemented using an electromagnetic cycle ergometer (KICKR Core trainer, Wahoo, USA) integrated within a large lower body pressure chamber (LBNP) (165 × 82 × 108 cm) designed for exercise echocardiography. The LBNP comprises an electric hydraulic jack that enables to select any degree from 0 to 45° of left lateral tilting. The combination of the left semilateral body position (17° relative to the horizontal) with lower body negative pressure allows for the simultaneous assessment of cardiac function (which requires a left lateral body position for high-quality and reproducible imaging) and aerobic capacity via the regulation of negative pressure inside the chamber (−50 mm Hg) to induce hemodynamic loads characteristic of the upright position, a physiological requirement to achieve VO_2peak_ [32, 33]. The test started with a warm-up period of 3 min at 20–30 W workloads. Thereafter, the workload was increased by 15–30 W every 60 s until exhaustion was reached in the recommended total duration of 7–10 min. O_2_ uptake and CO_2_ output were continuously measured and normalized by body weight (CardioCoach VO_2_, KORR Medical, USA). Calibration of the gas analyzers and the flowmeter was performed prior to each test. Breath-by-breath values were averaged over 15 s. The highest breath-by-breath average value was taken as VO_2peak_ provided that two of the following established criteria were fulfilled: plateau in O_2_ uptake despite increased workload, age-predicted HR_peak_ ± 10 bpm [34], respiratory exchange ratio ≥ 1.1 [10, 35].

### Statistical analysis

Statistical analysis was performed using SPSS 25.0 (SPSS, Chicago, IL). Data were tested for normal distribution with the Kolmogorov–Smirnov test and for homogeneity of variances with the Levene’s test. Comparisons among sex and age groups were performed via independent sample t tests. In addition, ANCOVA was used to determine the effects of sex and age on fundamental volumetric relationships in the circulatory system at peak exercise: (i) BV–LVEDV_peak_ and (ii) LVEDV_peak_–SV_peak_. A two-tailed *P*-value less than 0.05 was considered significant. All data are reported as mean (± SD).

## Results

### General characteristics, resting cardiac structure and function

Main general characteristics of the study participants are shown in Table 1. All individuals were non-smokers and non-obese (body mass index < 30 kg m^2^). MVPA levels were similar between sex and age groups (*P* ≥ 0.140). As expected, women presented lower anthropometric indices (height, weight, body surface area) compared with men (*P* ≤ 0.006), irrespective of the age group. No sex effect was found regarding resting blood pressure and HR (*P* ≥ 0.302). Nonetheless, older men exhibited higher resting blood pressure (*P* = 0.014) and older women lower resting HR (*P* = 0.044) compared with younger sex-matched counterparts. Regarding the cardiac phenotype at rest, sex effects were detected in young individuals, women having smaller LV volumes and SV than men (*P* ≤ 0.039). Likewise, age effects were noted in the LV, older men presenting lower LVEDV, SV and *Q* compared with young men (*P* ≤ 0.038). Furthermore, age per se reduced early peak diastolic inflow and annular velocities (*P* < 0.001), whereas late peak diastolic inflow and annular velocities were increased with older age (*P* = 0.001). Accordingly, the *E*/*A* velocity ratio was lower in older individuals, irrespective of sex (*P* < 0.001). Sex effects were specifically detected in older individuals, with women presenting higher tricuspid annular velocities than men (*P* ≤ 0.045). Furthermore, the myocardial IVCT (septal) was exclusively increased with older age in women (*P* = 0.001).

**Table 1:** General characteristics, resting cardiac structure and function

	Young			Older			*P* for age comparison	
			*P* for sex			*P* for sex		
*n*	14	14		29	28			
Age (year)	33.1 ± 6.0	28.5 ± 6.9	0.070	64.9 ± 7.3^a^	64.9 ± 6.4^a^	0.998	** < 0.001**	< 0.001
MVPA (h)	6.0 ± 3.3	8.5 ± 5.2	0.140	5.9 ± 2.7	6.2 ± 3.1	0.753	0.982	0.149
Height (cm)	169.0 ± 3.8	179.4 ± 9.9*	**0.001**	162.8 ± 7.9^a^	177.3 ± 5.6*	** < 0.001**	**0.008**	0.378
Weight (kg)	65.0 ± 5.9	78.7 ± 15.9*	**0.006**	60.4 ± 9.5	80.3 ± 9.3*	** < 0.001**	0.103	0.683
BMI (kg m^−2^)	22.8 ± 2.1	24.3 ± 3.6	0.170	22.7 ± 2.7	25.6 ± 2.8*	** < 0.001**	0.941	0.236
BSA (m^2^)	1.74 ± 0.08	1.97 ± 0.22*	**0.001**	1.64 ± 0.15^a^	1.98 ± 0.12*	** < 0.001**	**0.025**	0.968
SBP (mm Hg)	130.9 ± 16.2	132.8 ± 19.3	0.783	138.8 ± 32.2	147.2 ± 18.2^a^	0.233	0.393	**0.023**
DBP (mm Hg)	73.6 ± 20.6	73.8 ± 12.5	0.974	76.9 ± 18.5	78.4 ± 12.7	0.729	0.599	0.277
MBP (mm Hg)	88.0 ± 11.5	88.6 ± 18.0	0.918	96.2 ± 21.1	101.0 ± 12.9^a^	0.302	0.184	**0.014**
HR (bpm)	62.1 ± 9.2	59.4 ± 8.5	0.426	56.8 ± 7.2^a^	55.1 ± 5.9	0.342	**0.044**	0.061
Resting echocardiography								
RA (mL m^−2^)	21.00 ± 9.51	20.09 ± 5.52	0.761	17.94 ± 6.20	20.27 ± 6.78	0.180	0.211	0.932
RV EDA (cm^2^ m^−2^)	11.98 ± 2.42	11.53 ± 1.85	0.588	10.42 ± 2.04^a^	11.04 ± 2.30	0.284	**0.032**	0.491
RV ESA (cm^2^ m^−2^)	5.71 ± 1.21	4.90 ± 0.97	0.060	4.77 ± 1.71	5.22 ± 2.16	0.395	0.073	0.607
LA (mL m^−2^)	20.70 ± 8.40	21.55 ± 6.31	0.763	23.87 ± 10.57	22.41 ± 9.76	0.589	0.331	0.768
LVEDV (mL m^−2^)	45.48 ± 10.50	59.34 ± 14.22*	**0.007**	46.81 ± 9.06	50.06 ± 12.74^a^	0.270	0.671	0.038
LVESV (mL m^−2^)	12.38 ± 3.52	18.50 ± 6.89*	**0.006**	13.60 ± 5.36	15.29 ± 6.96	0.310	0.443	0.164
LVEF (%)	72.2 ± 7.9	69.1 ± 5.2	0.222	71.6 ± 7.7	70.4 ± 7.5	0.567	0.793	0.554
SV (mL m^−2^)	33.10 ± 9.67	40.62 ± 8.61*	**0.039**	33.21 ± 5.88	34.78 ± 7.57^a^	0.385	0.964	0.030
* Q* (L min^−1^ m^−2^)	2.02 ± 0.51	2.41 ± 0.60	0.075	1.99 ± 0.72	1.91 ± 0.44^a^	0.630	0.897	0.004
Diastolic function								
Tricuspid annular e' (cm s^−1^)	14.18 ± 4.29	12.10 ± 2.66	0.162	10.71 ± 1.81	9.11 ± 1.74	**0.005**	** < 0.001**	** < 0.001**
Tricuspid annular a' (cm s^−1^)	9.41 ± 2.06	9.00 ± 2.14	0.633	14.08 ± 2.51	12.49 ± 2.69	**0.046**	** < 0.001**	** < 0.001**
Tricuspid annular S' (cm s^−1^)	13.13 ± 2.57	12.81 ± 1.30	0.715	11.04 ± 1.51	11.07 ± 2.04	0.952	**0.015**	**0.017**
Mitral inflow E (cm s^−1^)	79.24 ± 12.69	74.83 ± 9.17	0.301	62.23 ± 12.04	54.90 ± 10.28	**0.019**	** < 0.001**	** < 0.001**
Mitral inflow A (cm s^−1^)	52.54 ± 10.74	45.29 ± 8.58	0.059	65.68 ± 12.11	60.20 ± 9.02	0.065	**0.001**	** < 0.001**
*E*/*A* ratio	1.55 ± 0.30	1.69 ± 0.30	0.213	0.957 ± 0.142	0.923 ± 0.183	0.451	** < 0.001**	** < 0.001**
Mitral annular e' (cm s^−1^)	14.59 ± 2.52	14.17 ± 2.91	0.683	9.90 ± 2.20	9.02 ± 1.82	0.120	** < 0.001**	** < 0.001**
Mitral annular a' (cm s^−1^)	8.44 ± 1.26	7.95 ± 1.65	0.393	11.52 ± 2.37	11.44 ± 1.95	0.886	** < 0.001**	** < 0.001**
*E*/*e*' ratio	5.59 ± 1.31	5.44 ± 1.09	0.752	6.53 ± 1.77	6.23 ± 1.38	0.496	0.083	0.073
IVRT (s)	0.120 ± 0.173	0.108 ± 0.159	0.859	0.074 ± 0.012	0.079 ± 0.015	0.204	0.402	0.513
IVCT (s)	0.053 ± 0.008	0.059 ± 0.010	0.120	0.065 ± 0.011	0.061 ± 0.011	0.229	**0.001**	0.533

Significant* P* values (< 0.05) are highlighted in bold

Data are expressed as mean ± SD

BMI, body mass index; BSA, body surface area; DBP, diastolic blood pressure; HR, heart rate; IVCT, isovolumic contraction time; IVRT, isovolumic relaxation time; LA, left atria; LVEDV, left ventricular end-diastolic volume; LVEF, left ventricular ejection fraction; MBP, mean blood pressure; MVPA, moderate-to-vigorous physical activity; *Q*, cardiac output; RA, right atria; RV EDA, right ventricular end-diastolic area; RV ESA, right ventricular end-systolic area; SBP, systolic blood pressure; SV, stroke volume

*P < 0.05 compared with age-matched women

^a^*P* < 0.05 compared with sex-matched young individuals

### Blood O_2_ carrying capacity and intravascular volumes

Table 2 presents key hematological determinants of cardiorespiratory fitness. According to established sex differences, blood O_2_ carrying capacity, as represented by Hb concentration and Hb_mass_, was markedly lower in women compared with men, irrespective of the age group (*P* < 0.001). An age effect was observed for Hb_mass_ per kg of body weight, which was reduced with older age in men (*P* ≤ 0.002). With respect to intravascular volumes, all of them (RBCV, PV, BV) were lower in absolute terms in women relative to men, in both young an older age groups (*P* ≤ 0.016). When normalized by body weight, RBCV remained lower in young and older women compared with age-matched men (*P* < 0.001), concurring with sex differences in O_2_ carrying capacity. With respect to the influence of age, total BV per kg of body weight was reduced in older compared with younger men (*P* = 0.018).

**Table 2:** Blood hemoglobin and intravascular volumes

	Young			Older			*P* for age comparison	
			*P* for sex			*P* for sex		
Hb_mass_ (g)	655.8 ± 86.6	1011.9 ± 133.8*	** < 0.001**	610.3 ± 81.3^a^	923.8 ± 116.0*^a^	** < 0.001**	**0.046**	**0.033**
Hb_mass_ (g kg^−1^)	10.3 ± 1.5	13.1 ± 1.5*	** < 0.001**	10.2 ± 1.2	11.6 ± 1.3*^a^	** < 0.001**	0.833	**0.002**
Hb (g dL^−1^)	13.2 ± 1.0	15.4 ± 0.7*	** < 0.001**	13.3 ± 0.6	14.9 ± 1.0*	** < 0.001**	0.512	0.111
Hct (%)	40.4 ± 3.0	47.2 ± 1.9*	** < 0.001**	40.9 ± 1.8	45.7 ± 3.1*	** < 0.001**	0.508	0.107
RBCV (mL)	2248 ± 295	3407 ± 450*	** < 0.001**	2060 ± 274^a^	3111 ± 387*^a^	** < 0.001**	**0.046**	**0.033**
RBCV (mL kg^−1^)	34.8 ± 5.2	43.9 ± 5.1*	** < 0.001**	34.5 ± 4.2	38.9 ± 4.3*^a^	** < 0.001**	0.837	**0.002**
PV (mL)	3332 ± 508	3810 ± 470*	**0.016**	2983 ± 418^a^	3709 ± 530*	** < 0.001**	**0.021**	0.550
PV (mL kg^−1^)	51.4 ± 7.6	49.3 ± 6.9	0.445	49.8 ± 6.1	46.4 ± 6.1	0.038	0.469	0.176
BV (mL)	5580 ± 717	7216 ± 882*	** < 0.001**	5043 ± 667^a^	6820 ± 796*	** < 0.001**	**0.020**	0.150
BV (mL kg^−1^)	86.2 ± 11.5	93.2 ± 11.5	0.118	84.3 ± 9.8	85.4 ± 8.8^a^	0.675	0.582	**0.018**

Significant* P* values (< 0.05) are highlighted in bold

Data are expressed as mean ± SD

BV, blood volume; Hb, hemoglobin concentration; Hb_mass_, total circulating hemoglobin mass; Hct, hematocrit; PV, plasma volume; RBCV, red blood cell volume

*P < 0.05 compared with age-matched women

^a^*P* < 0.05 compared with sex-matched young individuals

### Sex and age effects at peak exercise

The hematological, hemodynamic, cardiac and pulmonary variables at peak incremental exercise are presented in Table 3. Similar to resting values, blood O_2_ carrying capacity at peak exercise was lower in young and older women compared with age-matched men (*P* < 0.001). Peak blood pressures were similar between sexes (*P* ≥ 0.075), whereas SBP_peak_ and MBP_peak_ were higher with older versus younger age in women and men (*P* ≤ 0.018). Higher SVRpeak levels were observed with both female sex (*P* ≤ 0.038) and older age (*P* ≤ 0.010). Moreover, HR_peak_ was markedly reduced with older age (*P* < 0.001). As regards cardiac structure and function, LVEDV_peak_, SVpeak and *Q*_peak_ were lower in women compared with men irrespective of the age group (P ≤ 0.046). Furthermore, older age in women and men was associated with reduced *Q*_peak_ (*P* ≤ 0.022), primarily explained by lower HR_peak_ since SV_peak_ was not affected by older age (*P* ≥ 0.595). Absolute peak power output was lower in women compared with men in both age groups (206 ± 34 vs. 308 ± 58 W, *P* < 0.001 (young); 156 ± 46 vs. 197 ± 46 W, *P* ≤ 0.046 (older)) and reduced with older age in both sex groups (*P* < 0.001). Finally, cardiorespiratory fitness, as determined by VO_2peak_ (mL min^−1^ kg^−1^), was reduced with older age in both sexes (*P* < 0.001).

**Table 3:** Hematological, hemodynamic, cardiac and pulmonary variables at peak exercise

	Young			Older			*P* for age comparison	
			*P* for sex			*P* for sex		
Peak blood O2 carrying capacity								
Hb_peak_ (g dL^−1^)	14.1 ± 1.2	16.9 ± 0.8*	** < 0.001**	14.4 ± 0.8	15.9 ± 0.9*	** < 0.001**	0.371	**0.002**
Hct_peak_ (%)	43.1 ± 3.7	51.8 ± 2.5*	** < 0.001**	44.1 ± 2.3	48.8 ± 2.9*	** < 0.001**	0.382	**0.003**
Peak hemodynamics								
SBP_peak_ (mm Hg)	174.9 ± 33.2	185.0 ± 49.3	0.587	227.1 ± 52.3^a^	231.1 ± 48.5^a^	0.785	**0.005**	**0.018**
DBP_peak_ (mm Hg)	115.9 ± 40.9	85.7 ± 31.2	0.075	114.2 ± 29.4	115.1 ± 31.3^a^	0.918	0.894	**0.019**
MBP_peak_ (mm Hg)	109.6 ± 27.1	111.0 ± 35.1	0.917	144.8 ± 35.2^a^	148.7 ± 35.2^a^	0.713	**0.006**	**0.008**
SVR_peak_ (dyn s cm^−5^)	810.1 ± 272.3	571.8 ± 209.4*	**0.038**	1487.1 ± 796.6^a^	1004.7 ± 268.2*^a^	**0.010**	**0.010**	** < 0.001**
HR_peak_ (bpm)	168.8 ± 9.9	179.9 ± 9.41*	**0.005**	146.4 ± 12.3^a^	144.5 ± 13.5^a^	0.586	** < 0.001**	** < 0.001**
Peak echocardiography								
RA_peak_ (mL m^−2^)	14.52 ± 4.06	13.31 ± 4.04	0.437	12.77 ± 5.03	15.99 ± 7.55	0.066	0.267	0.223
RV EDA_peak_ (cm^−2^ m^2^)	9.50 ± 1.72	10.54 ± 1.96	0.146	8.76 ± 1.85	10.72 ± 2.32*	**0.001**	0.222	0.801
RV ESA_peak_ (cm^2^ m^−2^)	3.94 ± 0.88	4.31 ± 0.90	0.283	3.66 ± 0.84	4.14 ± 1.44	0.138	0.315	0.687
LA_peak_ (mLl m^−2^)	13.82 ± 5.08	12.60 ± 2.65	0.433	17.53 ± 6.83	18.75 ± 9.67^a^	0.583	0.079	**0.025**
LVEDV_peak_ (mL m^−2^)	45.12 ± 10.25	54.10 ± 9.84*	**0.026**	45.34 ± 8.97	55.23 ± 14.76*	**0.004**	0.944	0.799
LVESV_peak_ (mL m^−2^)	8.24 ± 2.10	9.73 ± 2.45	0.095	9.80 ± 3.53	11.25 ± 4.32	0.170	0.135	0.232
LVEFpeak (%)	81.5 ± 3.2	81.51 ± 4.92	0.983	78.6 ± 5.9	79.6 ± 6.1	0.548	0.090	0.306
SV_peak_ (mL m^−2^)	36.90 ± 8.98	44.36 ± 9.81*	**0.046**	35.54 ± 7.20	43.97 ± 12.54*	**0.003**	0.595	0.921
*Q*_peak_ (L min^−1^ m^−2^)	6.17 ± 1.26	7.99 ± 1.86*	**0.006**	5.19 ± 1.09^a^	6.39 ± 2.12*^a^	**0.011**	**0.012**	**0.022**
Peak pulmonary variables								
VO_2peak_ (mL min^−1^)	2699.7 ± 552.4	3826.9 ± 829.6*	** < 0.001**	1818.0 ± 498.8^a^	2607.4 ± 514.9*^a^	** < 0.001**	** < 0.001**	** < 0.001**
VO_2peak_ (mL min^−1^ kg^−1^)	41.7 ± 8.6	49.5 ± 11.5	0.051	30.3 ± 7.4^a^	32.9 ± 7.6^a^	0.186	** < 0.001**	** < 0.001**
Ve_peak_ (L min^−1^)	56.8 ± 28.9	69.4 ± 38.6	0.337	58.3 ± 21.5	70.8 ± 30.8	0.081	0.857	0.900

Significant* P* values (< 0.05) are highlighted in bold

Data are expressed as mean ± SD

DBP_peak_, peak diastolic blood pressure; Hb_peak_, peak hemoglobin concentration; Hct_peak_, peak hematocrit; HR_peak_, peak heart rate; LA_peak_, peak left atria; LVEDV_peak_, peak left ventricular end-diastolic volume; LVEF_peak_, peak left ventricular ejection fraction; MBP_peak_, peak mean blood pressure; O_2_, oxygen; *Q*_peak_, peak cardiac output; RA_peak_, peak right atria; RV EDA_peak_, peak right ventricular end-diastolic area; RV ESA_peak_, peak right ventricular end-systolic area; SBP_peak_, peak systolic blood pressure; SV_peak_, peak stroke volume: SVR_peak_, peak systemic vascular resistance; VO_2peak_, peak oxygen uptake

*P < 0.05 compared with age-matched women

^a^*P* < 0.05 compared with sex-matched young individuals

### Sex and age effects at a fixed submaximal workload

Table 4 displays the hemodynamic, cardiac and pulmonary variables at a given absolute submaximal workload (100 W). Submaximal blood pressures did not differ between sexes (*P* ≥ 0.257). With older age, SBP_100W_ and MBP_100W_ were elevated in women and men (*P* ≤ 0.006). Likewise, SVR_100W_ was augmented with older age irrespective of sex (*P* ≤ 0.004). In contrast, HR_100W_ was only affected by sex, with young and older women presenting higher values than age-matched men (*P* ≤ 0.013). Sex effects were detected regarding submaximal cardiac variables. Higher right atrial and ventricular dimensions at diastole were noted in older men compared with age-matched women (*P* ≤ 0.005). Moreover, LVEDV_100W_ was elevated in both older and young men relative to age-matched women (*P* ≤ 0.049). A sex effect in SV_100W_ reached significance in younger men, who presented higher SV_100W_ than age-matched women (*P* = 0.019). No effects of age were observed for submaximal cardiac structure and function (*P* ≥ 0.108). Concerning submaximal pulmonary variables, absolute O_2_ consumption was not affected by sex or age (*P* ≥ 0.535), whereas ventilation was augmented with older age in women (*P* = 0.033).

**Table 4:** Hemodynamic, cardiac and pulmonary variables at a fixed submaximal workload (100 W)

	Young			Older			*P* for age comparison	
			*P* for sex			*P* for sex		
Submaximal hemodynamics								
SBP_100W_ (mm Hg)	159.0 ± 21.2	157.2 ± 34.3	0.878	218.6 ± 48.9^a^	205.3 ± 39.5^a^	0.318	** < 0.001**	** < 0.001**
DBP_100W_ (mm Hg)	96.6 ± 27.3	90.5 ± 19.5	0.536	111.4 ± 24.9	103.5 ± 21.1	0.257	0.124	0.084
MBP_100W_ (mm Hg)	107.4 ± 13.8	109.5 ± 23.0	0.791	143.2 ± 31.3^a^	135.0 ± 24.9^a^	0.337	** < 0.001**	**0.006**
SVR_100W_ (dyn s cm^−5^)	1137.7 ± 246.7	1011.6 ± 310.1	0.283	1923.5 ± 712.6^a^	1573.8 ± 591.5^a^	0.079	** < 0.001**	**0.004**
HR_100W_ (bpm)	123.5 ± 20.1	105.6 ± 14.9*	**0.013**	121.0 ± 15.0	102.2 ± 11.3*	** < 0.001**	0.655	0.415
Submaximal echocardiography								
RA_100W_ (mL m^−2^)	16.10 ± 7.50	16.74 ± 5.56	0.801	13.41 ± 3.92	17.49 ± 6.01*	**0.005**	0.137	0.701
RV EDA_100W_ (cm^2^ m^−2^)	10.05 ± 2.77	11.17 ± 1.98	0.233	9.07 ± 2.05	10.72 ± 1.67*	**0.002**	0.207	0.451
RV ESA_100W_ (cm^2^ m−^2^)	4.42 ± 1.40	4.47 ± 0.89	0.910	3.77 ± 1.10	4.06 ± 1.20	0.345	0.108	0.266
LA_100W_ (mL m^−2^)	15.07 ± 7.40	15.53 ± 7.18	0.870	17.09 ± 6.23	19.98 ± 7.89	0.142	0.362	0.085
LVEDV_100W_ (mL m^−2^)	46.16 ± 7.91	56.43 ± 9.32*	**0.004**	43.03 ± 8.87	49.87 ± 15.19*	**0.049**	0.274	0.149
LVESV_100W_ (mL m^−2^)	10.22 ± 2.39	13.51 ± 2.98*	**0.003**	10.05 ± 3.99	12.48 ± 6.64	0.108	0.882	0.586
LVEF_100W_ (%)	77.9 ± 3.4	75.8 ± 5.2	0.229	77.1 ± 6.1	75.6 ± 8.8	0.466	0.662	0.918
SV_100W_ (mL m^−2^)	35.90 ± 6.41	42.91 ± 8.36*	**0.019**	32.98 ± 6.61	37.39 ± 11.62	0.093	0.185	0.123
Q_100W_ (L min^−1^ m^−2^)	4.37 ± 0.70	4.51 ± 0.92	0.656	3.95 ± 0.73	3.85 ± 1.37	0.724	0.082	0.109
Submaximal pulmonary variables								
VO_2,100 W_ (mL min^−1^)	1395.1 ± 331.7	1349.4 ± 359.0	0.760	1314.7 ± 355.8	1376.2 ± 367.1	0.535	0.524	0.838
VO_2,100 W_ (mL min^−1^ kg−^1^)	21.4 ± 4.5	17.1 ± 4.0*	**0.028**	22.2 ± 6.2	17.5 ± 5.1*	**0.003**	0.695	0.837
Ve_100W_ (L min^−1^)	21.9 ± 9.0	20.6 ± 15.1	0.801	33.0 ± 15.4^a^	28.8 ± 13.9	0.293	**0.033**	0.117

Significant* P* values (< 0.05) are highlighted in bold

Data are expressed as mean ± SD

DBP_100W_, submaximal (100 W) diastolic blood pressure; HR_100W_, submaximal (100 W) heart rate; LA_100W_, submaximal (100 W) left atria; LVEDV_100W_, submaximal (100 W) left ventricular end-diastolic volume; LVEF_100W_, submaximal (100 W) left ventricular ejection fraction; MBP_100W_, submaximal (100 W) mean blood pressure; Q_100W_, submaximal (100 W) cardiac output; RA_100W_, submaximal (100 W) right atria; RV EDA_100W_, submaximal (100 W) right ventricular end-diastolic area; RV ESA_100W_, submaximal (100 W) right ventricular end-systolic area; SBP_100W_, submaximal (100 W) systolic blood pressure; SV_100W_, submaximal (100 W) stroke volume: SVR_100W_, submaximal (100 W) systemic vascular resistance; Ve_100W_, submaximal (100 W) ventilation; VO_2,100W_, submaximal (100 W) oxygen uptake

*P < 0.05 compared with age-matched women

^a^*P* < 0.05 compared with sex-matched young individuals

### Sex and age effects on cardiovascular volumetric relationships

**Fig. 1:**
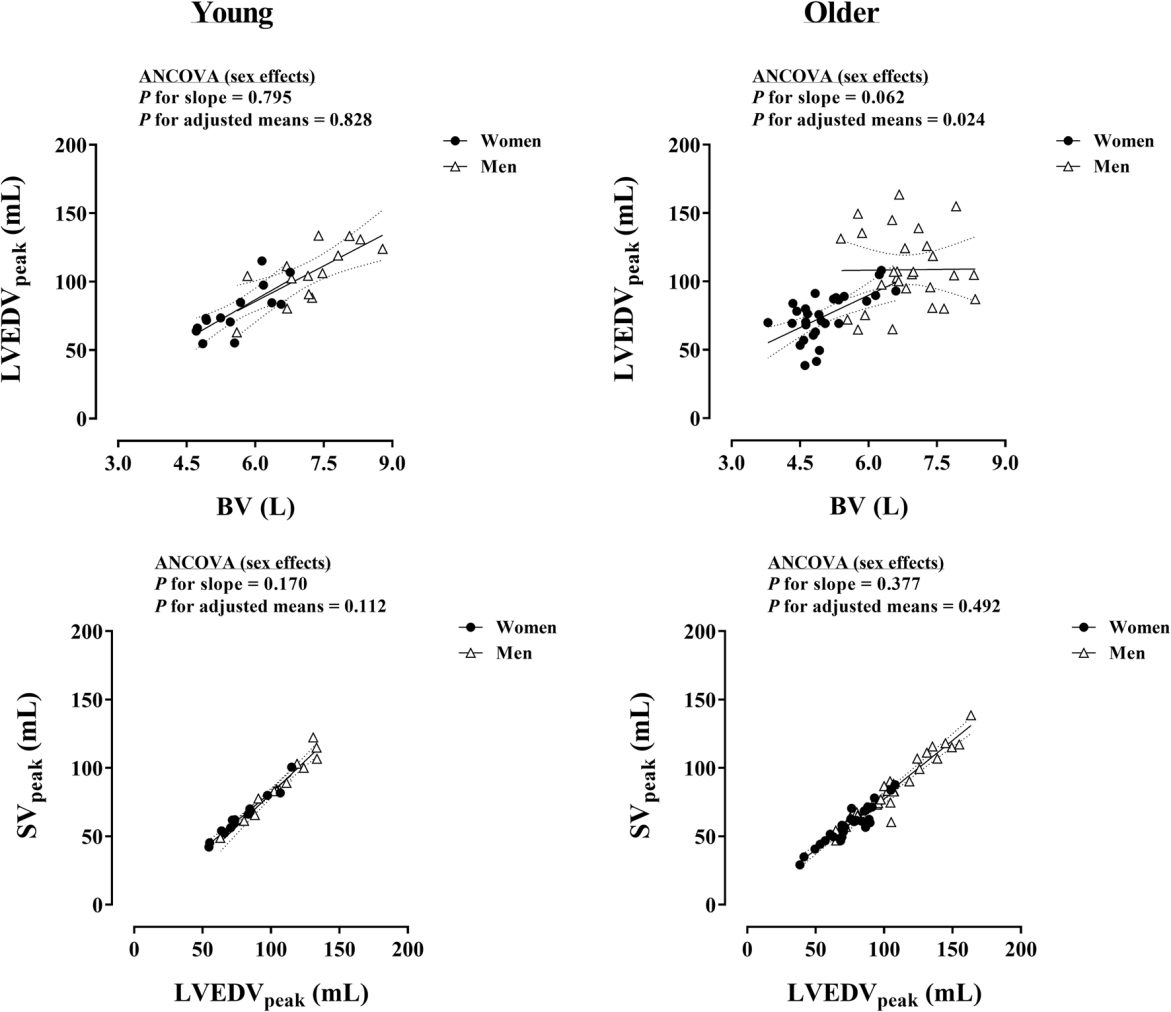
Influence of sex on BV–LVEDV_peak_ and LVEDV_peak_–SV_peak_ relationships in young and older individuals. Each graph includes the lines of best fit (95% confidence interval). BV, blood volume; LVEDV_peak_, left ventricular end-diastolic volume; SV_peak_, peak stroke volume

**Fig. 2:**
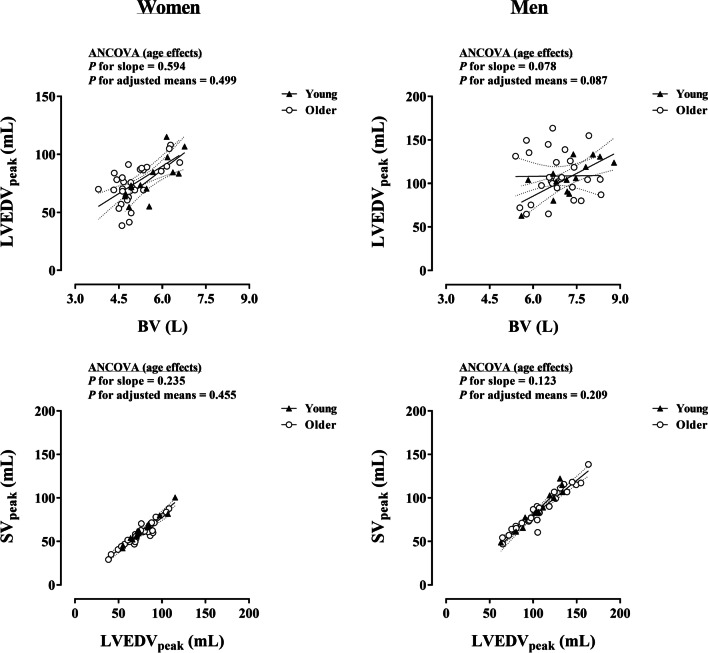
Influence of age on BV–LVEDV_peak_ and LVEDV_peak_–SV_peak_ relationships in women and men. Each graph includes the lines of best fit (95% confidence interval). BV, blood volume; LVEDV_peak_, left ventricular end-diastolic volume; SV_peak_, peak stroke volume

Figures 1 and 2 present the effects of sex and age, respectively, on fundamental volumetric relationships in the circulatory system, i.e., BV-LVEDV and LVEDV-SV at peak exercise. The slopes of BV-LVEDV_peak_ and LVEDV_peak_-SV_peak_ did not differ according to sex in each age group (*P* ≥ 0.062), thus the analysis of the effect of sex on adjusted means was subsequently performed (Fig. 1). A unique sex effect was detected for the adjusted means analysis of BV-LVEDV_peak_ in older individuals, demonstrating a lower LVEDV_peak_ for a given BV in women relative to men (*P* = 0.024). With respect to the effect of age, the slopes of BV–LVEDV_peak_ and LVEDV_peak_–SV_peak_ were similar in young and older individuals, irrespective of sex (*P* ≥ 0.078) (Fig. 2). The subsequent analysis of adjusted means in each sex group did not detect age effects (*P* ≥ 0.087).

## Discussion

The main purpose of this study was to assess the influence of sex and age on key physiological determinants of cardiorespiratory fitness. To this end, hematological, cardiac, hemodynamic and O_2_ uptake variables at rest and during exercise were analyzed in a cohort of healthy young and older women and men presenting with similar levels of physical activity. The major findings are: (1) BV adjusted by body weight do not differ between sexes; (2) women present smaller LV dimensions and output at peak exercise compared with men, regardless of age and sex differences in body size; (3) for a given absolute BV, older women exhibit lower LVEDV_peak_ than age-matched men. The present findings, along with those of ancillary nature that are conjointly developed hereunder, denote the interactive effects of sex and age on the fundamental basis of the circulatory system at peak working aerobic capacity.

The capacity to deliver O_2_ through the circulatory system, i.e., the main determinant of cardiorespiratory fitness [9, 10, 13], shows great quantitative variability in humans. Both genotypic and phenotypic variation contribute to the wide range of cardiac and aerobic capacities across the lifespan [9]. Beyond the manifest alterations induced by certain cardiovascular and hematological conditions [17, 21, 22], sex, age and physical activity levels are well-known to independently modulate key limiting factors of the O_2_ transport chain [20]. In this respect, human experimental investigations have demonstrated that not all steps of the O_2_ cascade from the atmosphere to the systemic circulation have the same importance [10, 36]. Lung structural and functional characteristics, which could in principle limit O_2_ transport, are overbuilt in relation to the potential delivery of O_2_ via the systemic circulation in healthy individuals exercising in normoxic conditions [37]. In men and presumably in women, VO_2peak_ is primarily a function of the heart’s ability to pump blood and arterial O_2_ content [9, 10, 13]. The latter factor is determined by a relatively fixed and sex-specific hematological variable, i.e., blood O_2_ carrying capacity [24]. In contrast, cardiac pumping capacity is primarily subordinated to the filling level of the circulatory system, a rather modifiable variable [6, 13]. The observation of similar BV per kg of body weight between sexes, notably in older individuals, yet substantially lower SV_peak_ in women versus men even after taking into account anthropometric differences suggests a potential novel sex dimorphism in a fundamental circulatory relationship [13–15]. In both young and older individuals, women exhibit nearly one-fifth lower SVpeak that is paralleled by a commensurate reduction in LVEDV_peak_ compared with men, i.e., the peak filling of the LV is substantially curtailed by the female sex. Integrative cardiovascular factors that may explain such a prominent sex dimorphism in the limitation of cardiorespiratory fitness are discussed hereunder.

The relationship between BV and LVEDV_peak_ reflects the extent to which the distribution of blood is optimized within the circulatory system at peak working capacity. In older individuals, LVEDV_peak_ per unit of BV was lower in women compared with men (Fig. 1). Sex or age effects were not observed in the relationship between LVEDV_peak_ and SV_peak_, indicating that the Frank–Starling mechanism was preserved. Thus, lower cardiac filling for a given BV may, at least in part, explain the reduced output (*Q*_peak_) in older women relative to age-matched men. Multiple cardiovascular regulatory mechanisms that primarily determine cardiorespiratory fitness might influence venous return and cardiac filling, ultimately limiting the systemic delivery of blood and thereby O_2_ [9]. These potential ‘weakest’ links can be in principle classified as belonging (intrinsic/central) or not (extrinsic/peripheral) to the anatomy and physiology of the heart. With respect to central factors, it is tempting to hypothesize a role for women’s predisposition to cardiac stiffness and impaired LV relaxation with advanced age [16, 21, 22, 38]. Women commonly present with augmented LV diastolic and systolic stiffness and steeper age-related increases in LV systolic stiffness compared with men [38]. Main LV functions may thus be prominently impaired in women with advance age, since older age per se (including men) did not alter the fundamental volumetric relationships (BV and LVEDV_peak_, LVEDV_peak_ and SVpeak) (Fig. 2). On the other hand, despite older age was associated with an impairment of common markers of LV diastolic function, they did not differ between sexes at rest (Table 1). Indeed, older women had similar mitral but higher tricuspid diastolic annular velocities at rest than age-matched men. While accurate non-invasive measures of diastolic function could not be obtained during exercise due time constraints for multiple cardiac imaging recordings at a given intensity, a prevailing association between resting and peak exercise diastolic function assessments has been found in catheterization studies [39, 40]. As regards peripheral factors, skeletal muscle arterial vasodilation is known to independently contribute to increase SV during exercise via the reduction of the systemic resistance to blood flow, i.e., SVR [41]. In this respect, we observed markedly augmented SVRpeak with female sex and older age. In addition, the myocardial isovolumic contraction time (IVCT) was increased with older age in women but not in men, which could also be partly related to female-specific LV systolic and central arterial stiffening [38]. Taken together, older women may have experienced the highest resistance to perfuse the systemic circulation at peak exercise, possibly resulting in lower output (*Q*_peak_), venous return and cardiac filling as a downstream consequence [42], which inverts the aforementioned causality sequence in the circulatory system [43]. Parenthetically, beyond the classic causality dilemma, whether a given factor within an intricate biological mechanism such as the circulatory system is conceptually considered as a cause or consequence depends on the standpoint. Experimental studies manipulating single variables at a time are needed to elucidate why older women exhibit higher SVR_peak_ and distribute their BV in a less optimal manner than opposite-sex counterparts. Such efforts might also shed light into resembling and highly prevalent pathophysiological alterations underlying deathly cardiac conditions in older women [21, 38, 44, 45].

A strong prognostic factor of cardiovascular health, *Q*_peak_, was independently modulated by sex and age [7, 8]. Specifically, both young and older women presented with reduced *Q*_peak_ compared with men matched by age, physical activity levels as well as aerobic capacity. In fact, notwithstanding the relevance of cardiac pumping capacity as a hallmark of cardiorespiratory fitness, its pulmonary correlate, i.e., VO_2peak_, did not differ between sexes. Likewise, despite cardiac output at a fixed submaximal workload (*Q*_100W_) was similar in women and men, submaximal O_2_ uptake (VO_2100W_) was higher in the former, regardless of age. Collectively considered, for a given unit of systemic blood flow (*Q*_peak_, *Q*_100W_), women extracted more O_2_ than men, albeit the female sex is typically characterized by reduced arterial O_2_ content owing to lower blood O_2_ carrying capacity throughout the adult lifespan [23], as herein demonstrated at rest and during exercise. The increased VO_2_/Q ratio in women may be underlined by central and peripheral factors. Starting from the periphery, recent studies have evidenced higher mitochondrial content and oxidative capacity, theoretically reflecting an enhanced potential for O_2_ extraction, in skeletal muscle fibers of women compared with men matched by VO_2peak_ [46–48]. Furthermore, the current observation of higher SVR_peak_ in women could also reflect an increased tightening of the systemic circulation leading to improved blood flow distribution and enhanced O_2_ extraction at peak exercise [49]. Yet, at a fixed submaximal workload another factor could be at play. Women have smaller conducting airways than men, also when adjusted by lung size [50]. Consequently, the female lung’s anatomy imposes greater mechanical resistance to breathing, particularly during exercise, thereby entailing a higher ventilatory work and O_2_ uptake (energy expenditure) for a given external work output [50]. The present findings therefore support the notion of women being less physiologically efficient than men, even when crucial counfounders are matched between sexes.

There are some limitations in this study that require comment. As an intrinsic limitation of cross-sectional analyses assessing the influence of sex and age, cause-and-effect relationships cannot be established, thus the conclusions should be taken with caution. Very long-term (lifelong) longitudinal studies, while seldom implemented due to outstanding logistical and technical challenges [51], could eventually provide a stronger level of evidence. Moreover, we selected healthy individuals in order to limit the influence of disease-related confounding factors. Further research will elucidate whether the present findings can be extrapolated to specific clinical conditions.

### Perspectives and significance

The current study assessed the impact of sex and age on major determinants of cardiorespiratory fitness in a cohort of healthy individuals matched by sex, age and physical activity levels. Following a comprehensive analysis at rest and during exercise, substantially smaller LV dimensions and correspondingly lower cardiac pumping capacity in women relative to men, independently of age and body size, are consistently demonstrated. Moreover, sex and age interact on a fundamental circulatory relationship, i.e., that of total circulating BV with LVEDV. Older women require higher BV for a certain level of LV filling, which suggests the presence of an intrinsic cardiovascular constraint, which concurs with functional features of prevalent life-threatening conditions in this population. Further experimental studies will ascertain whether such a circulatory limitation specific to older women can be overcome by tailored lifestyle and/or pharmacological interventions.

## Supplementary Information

**Additional file 1: Fig. S1.** Graphical outline of the study protocol. **Fig. S2.** Representative cardiac imaging during exercise according to the Simpson Method (biplane method of disks).

## Data Availability

All data generated or analyzed during this study are included in the manuscript.

## Abbreviations

BV: Blood volume
DBP: Diastolic blood pressure
Hb: Hemoglobin
Hb_mass_: Hemoglobin mass
Hct: Hematocrit
HR: Heart rate
LVEDV: Left ventricular end-diastolic volume
LVEF: Left ventricular ejection fraction
LVESV: Left ventricular end-systolic volume
MBP: Mean blood pressure
O_2_: Oxygen
PV: Plasma volume
*Q*: Cardiac output
RBCV: Red blood cell volume
SBP: Systolic blood pressure
SV: Stroke volume
SVR: Systemic vascular resistance
VO_2peak_: Peak oxygen uptake
