# Supplementary material for: Sex and age interaction in fundamental circulatory volumetric variables at peak working capacity
Source: Biol Sex Differ. 2022 Jan 3;13:1. doi: 10.1186/s13293-021-00409-9 (PMC8722064; doi:10.1186/s13293-021-00409-9)
Supplement: Supplementary file 1 — Additional file 1: Fig. S1. Graphical outline of the study protocol. Fig. S2. Representative cardiac imaging during exercise according to the Simpson Method (biplane method of disks). [file 13293_2021_409_MOESM1_ESM.docx]

**Additional file 1**

- **Figure S1.** Graphical outline of the study protocol.
- **Figure S2.** Representative cardiac imaging during exercise according to the Simpson Method (biplane method of disks).

**Figure S1.** Graphical outline of the study protocol.


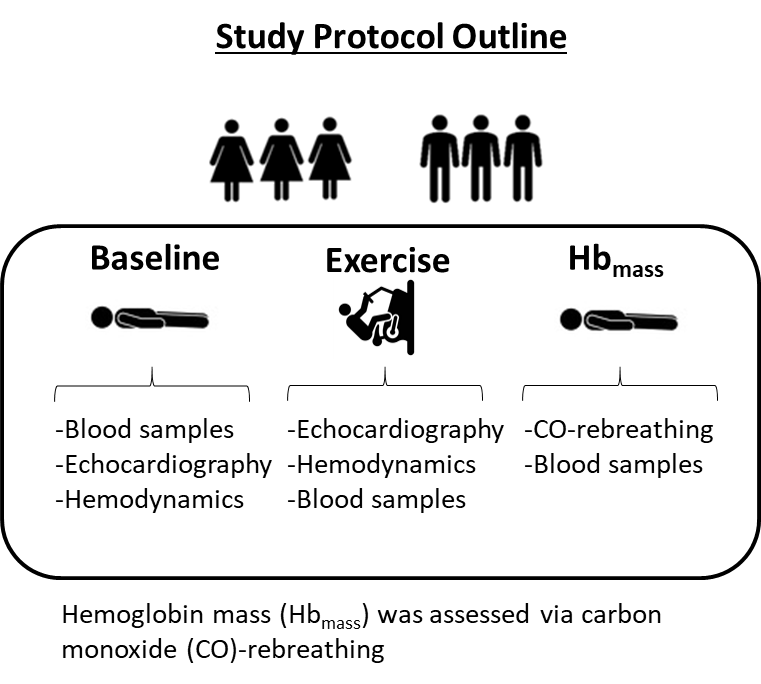


**Figure S2.** Representative image of cardiac imaging during exercise according to the Simpson Method (biplane method of disks).

**
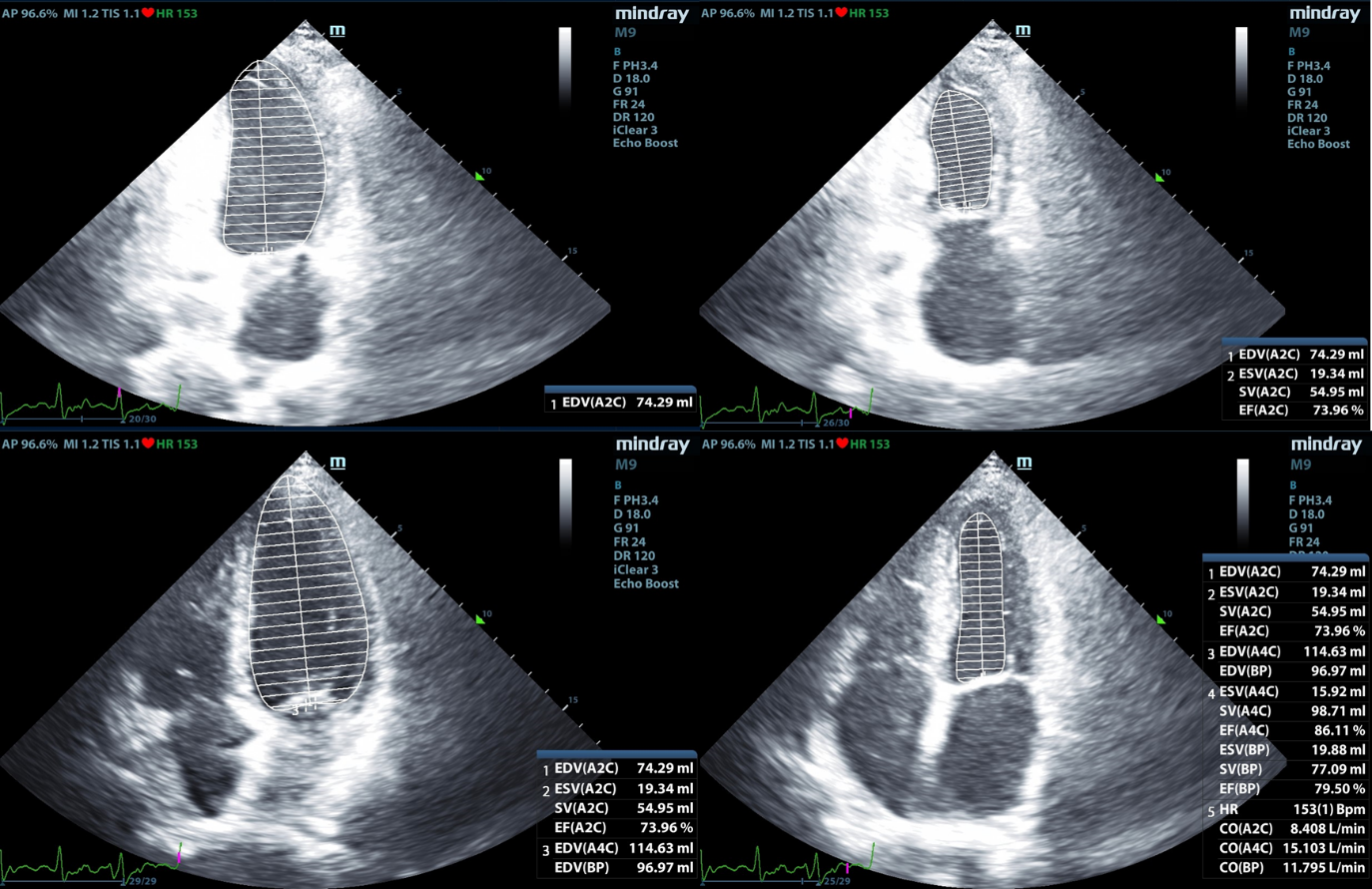
**

Following the American Society of Echocardiography and the European Association of Cardiovascular Imaging recommendations, cardiac chamber quantification was performed using the modified Simpson method (biplane method of disks) by tracing the endocardial border in both apical four-chamber and two-chamber views at end-diastole and end-systole [[1](#_ENREF_1), [2](#_ENREF_2)].

**References**

1. Lang RM, Badano LP, Mor-Avi V, Afilalo J, Armstrong A, Ernande L, et al. Recommendations for cardiac chamber quantification by echocardiography in adults: an update from the American Society of Echocardiography and the European Association of Cardiovascular Imaging. Eur Heart J Cardiovasc Imaging. 2015;16:3:233-70; doi:10.1093/ehjci/jev014.

2. Pellikka PA, Nagueh SF, Elhendy AA, Kuehl CA, Sawada SG, American Society of E. American Society of Echocardiography recommendations for performance, interpretation, and application of stress echocardiography. J Am Soc Echocardiogr. 2007;20:9:1021-41; doi:10.1016/j.echo.2007.07.003.
